# Data describing the relationship between world news and sustainable development goals

**DOI:** 10.1016/j.dib.2021.106978

**Published:** 2021-03-24

**Authors:** Gergely Honti, Tímea Czvetkó, Viktor Sebestyén, János Abonyi

**Affiliations:** a MTA-PE “Lendület” Complex Systems Monitoring Research Group, University of Pannonia, Veszprém, Hungary; b Institute of Advanced Studies Kőszeg, Chernel str. 14, H9730 Kőszeg, Hungary; c Sustainability Solutions Research Lab, University of Pannonia, Egyetem str. 10, H8200 Veszprém, Hungary

**Keywords:** Sustainable development goals (SDGs), News analysis, GDELT global knowledge graph, Network analysis

## Abstract

The data article presents a dataset and a tool for news-based monitoring of sustainable development goals defined by the United Nations. The presented dataset was created by structured queries of the GDELT database based on the categories of the World Bank taxonomy matched to sustainable development goals. The Google BigQuery SQL scripts and the results of the related network analysis are attached to the data to provide a toolset for the strategic management of sustainability issues. The article demonstrates the dataset on the 6th sustainable development goal (Clean Water and Sanitation). The network formed based on how countries appear in the same news can be used to explore the potential international cooperation. The network formed based on how topics of World Bank taxonomy appear in the same news can be used to explore how the problems and solutions of sustainability issues are interlinked.

**Specifications Table**

**Table:** 

Subject	Management, Monitoring, Policy and Law
Specific subject area	Dataset for news-based monitoring of sustainable development goals
Type of data	Statistics of sustainability news (Data table) Network for category analysis (Data table) Network for country co-occurrence analysis (Data table) World Bank taxonomy used for the categorization of the news mapped to sustainable development goals by experts (Data table) Google Big Query SQL code (Program Code)
How data were acquired	BigQuery SQL queries of the GDELT Global Knowledge Graph based on World Bank taxonomy matched to sustainable development goals
Data format	Raw data are in CSV format BigQuery SQL queries in plain text format Analyzed networks in Gephi format
Parameters for data collection	News appeared in 2019 and were categorised to topics of sustainability according to the World Bank taxonomy.
Description of data collection	Data is collected by running SQL queries, which are available on Mendeley [9]
Data source location	GDELT Global Knowledge Graph at Google Cloud contains categorised news from all of the countries of the world updated every 15 min (www.gdeltproject.org)
Data accessibility	The data is available on Mendeley [9] https://data.mendeley.com/datasets/xjzztkh7gt/1
Related research article	T. Czvetkó, G. Honti, V. Sebestyén, J. Abonyi. The intertwining of world news and Sustainable Development Goals: an effective monitoring tool, Heliyon, 7(2), 2021, doi: 10.1016/j.heliyon.2021.e06174 [7].

## Value of the Data

• The pre-processed and categorised data of the number and tone of the news can be used for strategic management and monitoring of sustainability issues.
• The dataset supports the comparison of the sensitivity of the media to sustainability issues in different countries.
• The network formed based on how countries appear in the same news can be used to explore the potential international cooperation.
• The network formed based on how topics of World Bank taxonomy appear in the same news can be used to explore how the problems and solutions of sustainability issues are interlinked.

## 1 Data Description

Sustainable development goals (SDGs) are the basic principles for achieving economic, industrial development goals while maintaining the ability to preserve the natural systems on which our civilizations depend. Therefore, monitoring and analyzing the multiple indices to intervene is critical. News play a significant part in conveying objectives and major focus areas of both governmental as well as public interests [1]. News analysis can play an active role in terms of defining the status of the roadmap for implementing the SDGs [2]. The localization of the SDGs is critical as social spaces are vital factors of the successful implementation and preservation of the goals [3]. GDELT uses natural language processing, data mining, and deep learning algorithms to extract and monitor news of the world. The Global Knowledge Graph (GKG) is a part of GDELT which records people, organizations, locations, themes and taxonomies, sources, tone and events of news into a network storage.

Included tables will be listed as: name of the table, description of the table, value of dataset, name of the column in the table, type of column and the description of the column.

- **world_bank_to_sdg.csv:** World Bank categories have been matched with the keywords of sustainable development goals. The matching was done manually by the authors who are experts of the field. One element can be matched to more sustainable development goals. All other results are dependent on this matching. This dataset provides the base for SQL Queries of the news as the World Bank identifier matches the category identifier in GDELT database.
  ○ WB_NUM (*int*) – World Bank identifier of the element. It matches the category identifier in the GDELT database.
  ○ SDG (*int*) – A sustainable development goal, numbered according to the UN declaration [4].
  ○ WB_SHORT (*string*) – World Bank taxonomy element identifier.
  ○ WB_NAME (*string*) – Full name of the topic based on World Bank classification.
  ○ WB_LVL (*int*) – Represents the ontological level of the World Bank identifier.
- **country_topic.csv**: Contains the country wise topics, average tone, total news count and the news percent of the topics. This dataset enables to create and analyze country profiles in regard to the sustainable development related news appearing.
  ○ COUNTRY (*string*) – Country identified with FIPS [5].
  ○ TOPIC (*string*) – World Bank taxonomy element.
  ○ NEWS_COUNT (*int*) – Count of topic matching articles.
  ○ TOTAL_NEWS_COUNT (*int*) – Number of total news in the country.
  ○ NEWS_PERCENT (*float*) – Percent of the matching articles.
  ○ LEVEL (*int*) – Level of taxonomy element.
- **country_topics_pivot.xlsx**:The pivot table of the news at different levels of the world bank ontology. This dataset shows the number of news stories appeared in a country and also measures its average tone.

The “PIVOT” contains the following information:

○ The first column represents the topics.
○ The last column represents the average tone of a topic across all countries.
○ The other columns are the enumerated countries, and the average tone of the category in the category according the topic.
○ The last row represents the average tone of the country according the selected topics.

- **news_analysis_countries_topics.csv**: The file contains the numeric aggregation of the news by countries. This dataset defines country profiles in regard to their attitude towards the sustainable development goals (tone of news – average, very positive, very negative) in counts and in percentage.
  ○ COUNTRY (*string*) – Country identified with ISO_A3.
  ○ TOPIC (*string*) – World Bank category.
  ○ NEWS_COUNT (*int*) – Count of the articles matching the category.
  ○ TOTAL_NEWS_COUNT (*int*) – Total number of articles in the country.
  ○ TONE (*float*) – Average tone of the articles in the country, which are matching the topic.
  ○ NEWS_PERCENT (*float*) – Percent of the news matching the topic in the selected country.
  ○ NEWS_POS_COUNT (*int*) – Very positive news count, based on the overall tone, where it is ≥ 10
  ○ NEWS_NEG_COUNT (*int*) – Very negative news count, based on the overall tone, where it is ≤ −10
  ○ NEWS_POS_PERCENT (*float*) – Very positive news in percent (NEWS_POS_COUNT / NEWS_COUNT)
  ○ NEWS_NEG_PRECENT (float) – Very negative news in percent (NEWS_ NEG _COUNT / NEWS_COUNT)
- **country_network.csv**: News category-based co-occurrences of the countries in sustainability news. This dataset is the base of a multilayered network representing countries co-occurrence in regard to a news category and its tone. The data table contains the fields described below:
  ○ Source (*string*) – A node, representing the source country encoded in ISO_A3.
  ○ Target (*string*) – A node, representing the target country encoded in ISO_A3.
  ○ Layer (*string*) – World Bank category in numeric format.
  ○ Layer Name (*string*) – World Bank category name in readable format.
  ○ Weight (*int*) – The weight of the described edge, representing the number of co-occurrences in the articles of the countries.
  ○ Weight_POS (*int*) – alternative edge description, representing the number of very positive co-occurrences of the countries, based on overall tone ≥ 10.
  ○ Weight_NEG (*int)* – alternative edge description, representing the number of very negative co-occurrences of the countries, based on overall tone ≤ −10.
- **country_network_SDG.csv**: SDG-based co-occurrences of countries in sustainability news. This dataset is the base of a multilayered network representing countries co-occurrence in regard to the sustainable development goals and their tone. The data table has the fields described below:
  ○ Source (*string*) – A node, representing the source country encoded in ISO_A3.
  ○ Target (*string*) – A node, representing the target country encoded in ISO_A3.
  ○ Layer (*int*) – Represents a sustainable development goal as a layer in the network.
  ○ Weight (*int*) – The weight of the described edge, representing the number of co-occurrences in the articles of the countries.
  ○ Weight_POS (*int*) – alternative edge description, representing the number of very positive co-occurrences of the countries, based on overall tone ≥ 10.
  ○ Weight_NEG (*int)* – alternative edge description, representing the number of very negative co-occurrences of the countries, based on overall tone ≤ −10.
- **wb1_un_net.csv**: Undirected network of World Bank topics on taxonomy level 1 and based on the United Nations My World Survey topics. This network shows how different topic occur together in the news. Networks can be formed based on the average tone and frequency of the co-occurrence of the topics.
  ○ Source (*string*) – Represents the source node topic. Eighter from the United Nations My World Survey topics or from the World Bank.
  ○ Target (*string*) – Represents the target node topic. Eighter from the United Nations My World Survey topics or from the World Bank.
  ○ AVG_TONE (*float*) – Represents the average tone between the topics.
  ○ Record_Count (*int*) – Represents the total co-occurring record count. Both nodes appearing same time in an article.
  ○ SOURCE_TOTAL_RECORD (*int*) – Total record count of the source node.
  ○ TARGET_TOTAL_RECORD (*int*) – Total record count of the target node.
  ○ SOURCE_POSNEWS_RECORD_COUNT (*int*) – Represents the total record count of positive news (tone ≥ 10) from the source category.
  ○ TARGET_POSNEWS_RECORD_COUNT (*int*) – Represents the total record count of positive news (tone ≥ 10) from the target category.
  ○ SOURCE_POSNEWS_PERCENT (*float*) – Shows the percentage of the positive news in the source topic.
  ○ TARGET_POSNEWS_PERCENT (*float*) – Shows the percentage of the positive news in the target topic.
  ○ SOURCE_NEGNEWS_RECORD_COUNT (*int*) – Represents the total record count of negative news (tone ≤ −10) from the source category.
  ○ TARGET_NEGNEWS_RECORD_COUNT (*int*) – Represents the total record count of negative news (tone ≤ −10) from the target category.
  ○ SOURCE_NEGNEWS_PERCENT (*float*) – Shows the percentage of the negative news in the source topic.
  ○ TARGET_NEGNEWS_PERCENT (*float*) – Shows the percentage of the negative news in the target topic.
- **world_bank_taxonomy.csv:** The file contains the whole World Bank taxonomy [6]. This dataset defines the categories of the news and their hierarch based on the World Bank taxonomy. The table includes the following fields:
  ○ WB_NUM (*int*) – World Bank identifier of the element. It matches the category identifier in the GDELT database.
  ○ Name (*string*) – The full name of the category.
  ○ Level (*int*) – Ontological level of the category. In World Bank database, there are five levels of the domains.
- **country_ISO_A3.csv**: A table for ISO_A3 codes. This dataset is used to encode countries.
  ○ ADMIN (*string*) – Country name.
  ○ ISO_A3 (*string*) – ISO_A3 encoding of the country.
- **country_FIPS.csv:** A lookup table for FIPS codes. To display data on a map, this separate data table shows the GPS coordinates of the countries.
  ○ FIPS (*string*) – FIPS encoding of the country.
  ○ ADMIN (*string*) – Country name.
  ○ LATITUDE (*float*) – Latitude of the middle of the country.
  ○ LONGITUDE (*float*) – Longitude of the middle of the country.

The data was generated by the following Google BigQuery SQL scripts that can be easily modified to study specific time periods. The SQL scripts are commented to highlight how the code can be tailored for specific analysis. All scripts have the following setting options:

○ TIMEFRAME_START (*datetime*) – Starting time
○ TIMEFRAME_END (*datetime*) – Ending time
○ TOPICS (*list*) – List for categories enumerated in this filter. The filter is an OR separated list, for the where statement.

- **1_query_fulldatabase.sql:** To query raw data, we recommend the following Google BigQuery script, which returns the articles, the related countries, topics and tone of news. Google BigQuery SQL script that returns the following data:
  ○ GKGRECORDID (*string*) – Unique identifier of an article
  ○ V2SOURCECOMMONNAME (*string*) – URL to the original medium published the article.
  ○ V2DOCUMENTIDENTIFIER (*string*) – Full URL to the article.
  ○ COUNTRYCODE (*string*) – Countries mentioned in the article.
  ○ THEME (*string*) – World Bank identifiers for the categories of the article.
  ○ TONE (*float*) – Overall tone of the article.
- **2_country_topic_statistics.sql:** To compile comprehensive statistics, this script is suggested to use. This Google BigQuery SQL script generates statistics of the selected themes by country and return the following values:
  ○ COUNTRY (*string*) – Countries mentioned in the article.
  ○ TOPIC (*string*) – World Bank identifiers for the categories of the article.
  ○ NEWS_COUNT (*int*) – The count of the articles, mentioning the country and the topic.
  ○ AVG_TONE (*float*) – Average tone of the articles in the category mentioning the country.
  ○ TOTAL_NEWS_COUNT (*int*) – The total count of the articles mentioning the country.
  ○ NEWS_PERCENT (*int*) – Percent of the category mentioning the country, according to the total news count of the country.
  ○ POSITIVE_NEWS_COUNT (*int*) – Number of the positive articles (overall tone ≥ 10) in the topic.
  ○ NEGATIVE_NEWS_COUNT (*int*) - Number of the negative articles (overall tone ≤ −10) in the topic.
  ○ POSITIVE_NEWS_PERCENT (*float*) – Percent of the positive articles.
  ○ NEGATIVE_NEWS_PERCENT (*float*) - Percent of the negative articles.
- **3_country_network_incl_stat.sql:** News articles often refer to multiple countries, therefore to display the link between countries, we recommend to use this Google BigQuery SQL script that creates the network of countries and return the following values:
  ○ Source (*string*) – Country representing the source node in the network.
  ○ Target (string) – Country topic representing the target node in the network.
  ○ AVG_TONE (*float*) – Average tone between the two countries, based on the co-occurring documents.
  ○ Weight (*int*) – Represents the number of co-occurrences of the topics.
  ○ SOURCE_TOTAL_RECORD (*int*) – Represents the total number of occurrences of the source node.
  ○ TARGET_TOTAL_RECORD (*int*) – Represents the total number of occurrences of the target node.
  ○ SOURCE_POSNEWS_RECORD_COUNT (*int*) – Represents the total number of very positive news occurrences of the source node.
  ○ TARGET_POSNEWS_RECORD_COUNT (*int*) – Represents the total number of very positive news occurrences of the target node.
  ○ SOURCE_POSNEWS_PERCENT (*float*) – The percent of the positive news of the source node.
  ○ TARGET_POSNEWS_PERCENT (*float*) – The percent of the positive news of the target node.
  ○ SOURCE_NEGNEWS_RECORD_COUNT (*int*) – Represents the total number of very negative news occurrences of the source node.
  ○ TARGET_NEGNEWS_RECORD_COUNT (*int*) – Represents the total number of very negative news occurrences of the target node.
  ○ SOURCE_NEGNEWS_PERCENT (*float*) – The percent of the negative news of the source node.
  ○ TARGET_NEGNEWS_PERCENT (*float*) – The percent of the negative news of the target node.
- **4_topic_network_incl_stat.sql:** To analyze the relationship between topics, this query is recommended to use as it creates the undirected network of topics and their statistical attributes. The following values are returned:
  ○ Source (*string*) – World Bank topic representing the source node in the network
  ○ Target (string) – World Bank topic representing the target node in the network
  ○ AVG_TONE (*float*) – Average tone between the two topics, based on the co-occurring documents.
  ○ Weight (*int*) – Represents the number of co-occurrences of the topics.
  ○ SOURCE_TOTAL_RECORD (*int*) – Represents the total number of occurrences of the source node.
  ○ TARGET_TOTAL_RECORD (*int*) – Represents the total number of occurrences of the target node.
  ○ SOURCE_POSNEWS_RECORD_COUNT (*int*) – Represents the total number of very positive news occurrences of the source node.
  ○ TARGET_POSNEWS_RECORD_COUNT (*int*) – Represents the total number of very positive news occurrences of the target node.
  ○ SOURCE_POSNEWS_PERCENT (*float*) – The percent of the positive news of the source node.
  ○ TARGET_POSNEWS_PERCENT (*float*) – The percent of the positive news of the target node.
  ○ SOURCE_NEGNEWS_RECORD_COUNT (*int*) – Represents the total number of very negative news occurrences of the source node.
  ○ TARGET_NEGNEWS_RECORD_COUNT (*int*) – Represents the total number of very negative news occurrences of the target node.
  ○ SOURCE_NEGNEWS_PERCENT (*float*) – The percent of the negative news of the source node.
  ○ TARGET_NEGNEWS_PERCENT (*float*) – The percent of the negative news of the target node.

Included Gephi files that contain the networks generated based on the presented data

- **topic_net_sdg6.gephi** – Contains the network of World Bank taxonomy elements from the SDG6 matching. It defines the connections (co-occurrence) of the SDG6-related WB taxonomy elements and their tone.
- **topic_net_all_sdg.gephi** – Contains the network of World Bank taxonomy elements from all SDGs matching. It defines the connections (co-occurrence) of the SDG-related WB taxonomy elements and their tone.
- **countries_net_sdg13.gephi** – Contains the network of the countries interconnected by the mentions about SDG13. It defines the connections (co-occurrence) of countries in regard to the SDG13-related news and their tone.

## 2 Experimental Design, Materials and Methods

Governmental policies are operated in long-term planning horizon by reflecting socioeconomic as well as environmental development focused visions. We aim to combine the advantage of the news analysis through The GDELT Project as well as the network analysis models to reveal interconnection.

The following steps of news-centered network analysis of sustainability issues can be distinguished:

- **Determination of search words connected to SDGs:** Search words are basis on the World Bank Group's Topical Taxonomy [5] and the My World 2015 [6] survey contributed to the formulation of Agenda 2030. The selection of search words and their association with the sustainable development goals (see table *world_bank_to_sdg.csv*) was done manually by the authors who are experts of the field, however, the methodology allows them to validate through the joint occurrences of the topics.
- **Development of the related SQL (Structured Query Language) Queries:** GDELT Global Knowledge Graph (GKG) database records people, organizations, locations, themes, taxonomies, sources, tone and events of news and makes this huge amount of data available as a quaryable dataset in the Google BigQuery (GBQ). Therefore, the systematic queries are based on a schematic SELECT query which captures the main details of the GKG database, namely location, date, topics and tone of news.
- **Generation of networks:** A labelled multilayer network is created, which enables to identify the profiles of countries or regions as well as examine their overreaching national relationships based solely on news appearing. The network is generated using GDELT geolocation, topic recognition and sentiment analysis. Based on the location mentioned in the article GDELT geolocates the articles to countries and cities. The multidimensional network can be defined as nodes (V), edges (E) and dimensions (D). The edge represents the connection between two nodes (u and v) in a dimension (d). It is express as the following:
  G=(V,E,D)
  E={(u,v,d),u,v∈V,d∈D}

Furthermore, the categories of the article define which layer the previously mentioned edge appears in. An article includes characteristics as article's id (i), publication date (t_i_), the identified set of locations mentioned within it (L_i_), the dimensions and tags of the article (D_i_) and also its sentiment (s_i_).

$$a_{i}=\langle i,t_{i},L_{i},D_{i},s_{i}\rangle$$

Two types of networks are built, one is when the nodes are the topics, the edges are the news and the dimension of the edges is the countries or groups of countries and the weight of the edges is the number of pieces and/or tone. The other option is when the nodes represent the countries and edges are the news of a topic and the weight of the edges can be determined from the number of articles.

This methodological development allows continuous monitoring throughout the world through online queries, therefore measuring the social acceptance of SDGs and encouraging participation in terms of their implementation, as well as helping countries around the world to share experiences concerning their problems and successes, which is essential for the implementation of the Agenda [7].

### 2.1 Validation of the applicability of the data

We categorized the news gained about according to the 17 sustainable development goals. In the following we will only consider for an example analysis the 6th goal of the UN sustainable development goals, which is the “Clean Water and Sanitation” goal. This covers 5 624 192 articles, from 226 countries.

The overview of the top 10 most mentioned countries by the news already creates an impression that this topic in these countries is fairly natural, tone is around 0, however, there are certain outliers in the number of positive and negative articles. An article is positive if the overall tone is above 10 and negative if it is below −10.

**Fig. 1:**
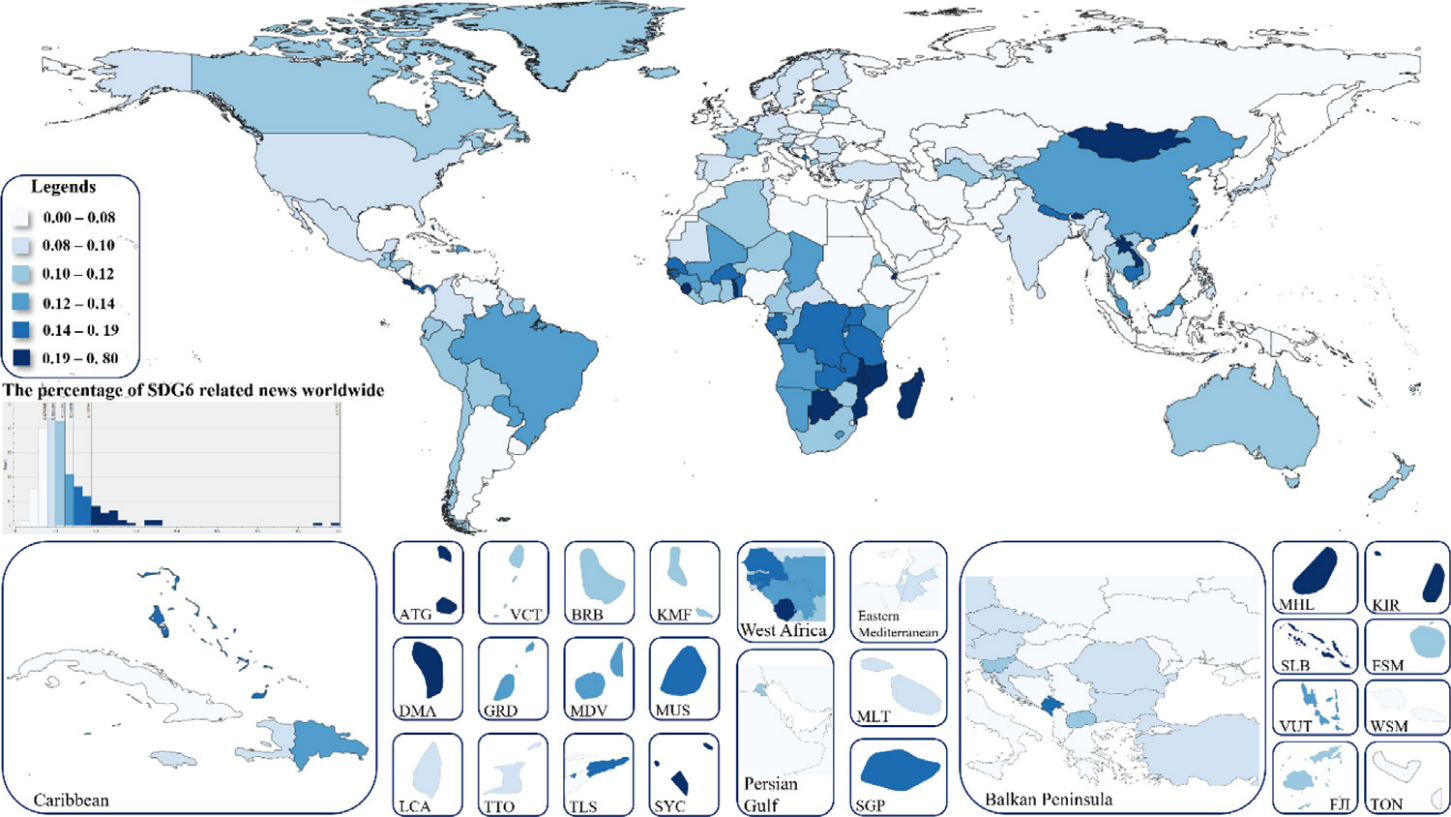
Choropleth of the SDG6 in the world. The darker color of the country represents more central Clean Water and Sanitation issue. The abbreviations and the modelled values of the cutouts are: Antigua and Barbuda ATG (0.201%), St. Vincent and the Grenadines VCT (0.110%), Barbados BRB (0.115%), Comoro Islands KMF (0.119%), Dominica DMA (0.284%), Grenada GRD (0.138%), Maldives MDV (0.135%), Mauritius MUS (0.157%), St. Lucia LCA (0.083%), Trinidad and Tobago TTO (0.093%), Timor-Leste TLS (0.181%), Seychelles SYC (0.225%), Malta MLT (0.099%), Singapore SGP (0.184%), Marshall Islands MHL (0.354%), Kiribati KIR (0.222%), Solomon Islands SLB (0.203%), Micronesia (Federated States of) FSM (0.113%), Vanuatu VUT (0.139%), Samoa WSM (0.061%), Fiji FJI (0.108%), Tonga TON (0.061%).

The distribution of global news related to SDG6 (Ensure availability and sustainable management of water and sanitation for all) is shown in Fig. 1. The map cutouts were selected based on the Global Burden of Disease Study by Fullman et al. [8].

Fig. 1 shows the darker countries are discussed the Clean Water and Sanitation topic more detailed. It can be seen, that in Norfolk Island (NFK) from the overall news is occupied 0,8% percent of the news related to SDG6. In Pitcairn Islands (PCN), the 0,75% of news talks about Clean Water and Sanitation.

**Fig. 2:**
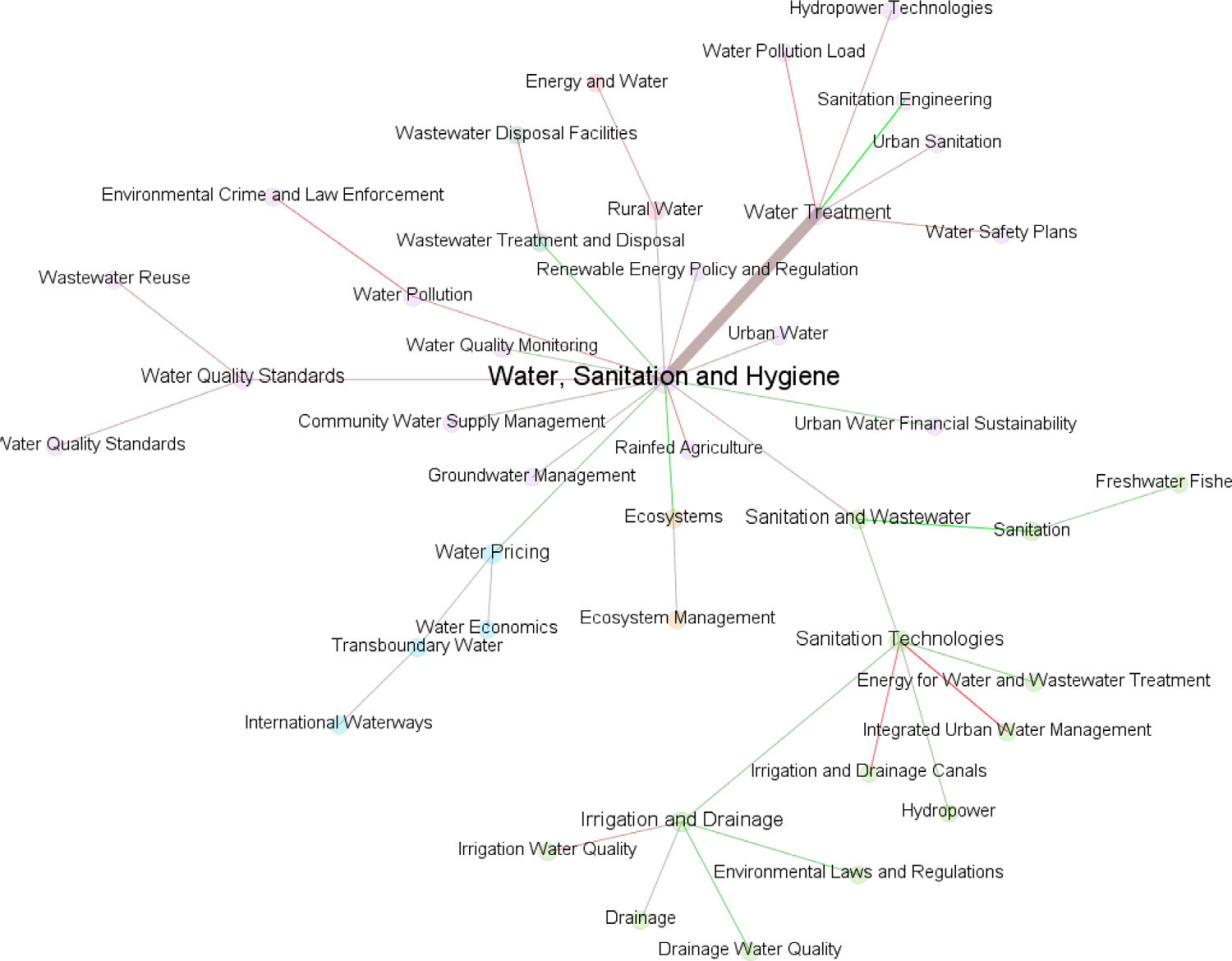
Network of the SDG6 related topics. The edges are colored with red if the sustainability news tends to be negative and green if the sustainability news tends to be positive. The edges are as thick as the normalized counts of articles between the categories. (For interpretation of the references to color in this figure legend, the reader is referred to the web version of this article.)

Fig. 2 shows the connection between the topics of SDG6. The edges are colored with red having bad tone when connecting two topics, and green with good tone connecting them. The thickness represents the number of connections between the topics. It can be seen, that “Water Treatment” and “Water, Sanitation and Hygiene” are the most common topics appearing together in the news, with an overall bad tone. While “Water, Sanitation and Hygiene” is connecting to Ecosystems with an overall good tone.

The ever increasing social participation and environmental awareness can be achieved primarily through news, therefore, their monitoring is essential. We presented a dataset that supports the news-based analysis of sustainable development goals. We have demonstrated the applicability of the data by presenting how the news related to the 6th SDG “Clean Water and Sanitation” goal are distributed among the countries and how the topics of World Bank taxonomy are connected to this critical issue.

Thanks to the intelligent functions behind the studied GDELT Knowledge Graph the proposed tool overcomes the limitations of geographical coverage and language barriers. With the proposed Google BigQuery SQL scripts the global media is discoverable and thematically analyzable in terms of the SDGs, which was not possible before.

## Ethics Statement

Not applicable.

## CRediT Author Statement

**Viktor Sebestyén:** Conceptualization, Validation, Investigation, Formal analysis, Writing Original Draft, Visualization; **Gergely Honti:** Software, Formal analysis, Data Curation, Writing - Original Draft; **Tímea Czvetkó:** Writing - Original Draft, Investigation, Validation; **János Abonyi:** Methodology, Validation, Resources, Writing - Review & Editing, Supervision, Project administration, Funding acquisition.

## Declaration of Competing Interest

The authors declare that they have no known competing financial interests or personal relationships which have, or could be perceived to have, influenced the work reported in this article.
